# Oxidative High Mobility Group Box-1 Accelerates Mitochondrial Transfer from Mesenchymal Stem Cells to Colorectal Cancer Cells Providing Cancer Cell Stemness

**DOI:** 10.3390/ijms26031192

**Published:** 2025-01-30

**Authors:** Rika Sasaki, Yi Luo, Shingo Kishi, Ruiko Ogata, Yukiko Nishiguchi, Takamitsu Sasaki, Hitoshi Ohmori, Rina Fujiwara-Tani, Hiroki Kuniyasu

**Affiliations:** 1 Department of Molecular Pathology, Nara Medical University, 840 Shijo-cho, Kashihara 634-8521, Nara, Japan; rika0st1113v726296v@icloud.com (R.S.); lynantong@hotmail.com (Y.L.); nmu6429@yahoo.co.jp (S.K.); pkuma.og824@gmail.com (R.O.); yukko10219102@yahoo.co.jp (Y.N.); takamitu@fc4.so-net.ne.jp (T.S.); brahmus73@hotmail.com (H.O.); rina_fuji@naramed-u.ac.jp (R.F.-T.); 2 Pathology Laboratory, Research Institute, Tokushukai Nozaki Hospital, 2-10-50 Tanigawa, Daito 574-0074, Osaka, Japan

**Keywords:** mitochondrial transfer, oxidized HMGB1, NF–κB, chemoresistance, cancer stemness, colorectal cancer

## Abstract

Mitochondria are important organelles for cell metabolism and tissue survival. Their cell-to-cell transfer is important for the fate of recipient cells. Recently, bone marrow mesenchymal stem cells (BM-MSCs) have been reported to provide mitochondria to cancer cells and rescue mitochondrial dysfunction in cancer cells. However, the details of the mechanism have not yet been fully elucidated. In this study, we investigated the humoral factors inducing mitochondrial transfer (MT) and the mechanisms. BM-MSCs produced MT in colorectal cancer (CRC) cells damaged by 5-fluorouracil (5-FU), but were suppressed by the anti-high mobility group box-1 (HMGB1) antibody. BM-MSCs treated with oxidized HMGB1 had increased expression of MT-associated genes, whereas reduced HMGB1 did not. Inhibition of nuclear factor–κB, a downstream factor of HMGB1 signaling, significantly decreased MT-associated gene expression. CRC cells showed increased stemness and decreased 5-FU sensitivity in correlation with MT levels. In a mouse subcutaneous tumor model of CRC, 5-FU sensitivity decreased and stemness increased by the MT from host mouse BM-MSCs. These results suggest that oxidized HMGB1 induces MTs from MSCs to CRC cells and promotes cancer cell stemness. Targeting of oxidized HMGB1 may attenuate stemness of CRCs.

## 1. Introduction

Colorectal cancer (CRC) ranks second and first in cancer mortality among Japanese men and women, respectively [1]. A five year survival rate for CRC patients is about 70% [1], with a particularly high mortality rate after relapse [2]. In patients with recurrent CRC, therapeutic agents targeting only tumor cells have limitations and new treatment strategies are needed. Tumor development and recurrence depend on the interaction of tumor cells and normal cells in the tumor microenvironment (TME) [3]. Within the TME, mesenchymal stem cells (MSCs) are recruited into the tumors by tumor-derived factors [4]. Moreover, physical contact between MSCs and tumor cells provides intercellular signaling, leading to tumor stromal formation and tumor cell growth [5]. Therefore, therapies targeting the interaction between MSCs and tumor cells may be relevant.

In recent years, many studies have elucidated the cell-to-cell interaction via mitochondrial migration between MSCs and tumor cells [6]. MSC-derived mitochondria migrate into tumor cells through tunneling nanotubes (TNT) by the actin filament-forming cytoskeletal system, which is named mitochondrial transfer (MT) [7]. MT occurs in many cancers [8], and it plays a role in cancer cell evasion from the immune system and provides drug resistance in tumor cells [9,10]. However, there are few studies on the mechanisms of MT between MSCs and CRC cells, and the impact of MT in CRCs remains unclear.

HMGB1 is released extracellularly from necrotic tumor cells and induces nuclear factor–κB (nuclear factor–kappa-light-chain-enhancer of activated B cells) activation and cytokine production via the receptor’s receptor for glycation end products (RAGE) and toll-like receptor 4 (TLR4) expressed on tumor cells, promoting tumor growth, survival, invasion and metastasis [11,12]. HMGB1 has three cysteine residues, Cys23, Cys45, and Cys106, and modifications of these cysteines determine the biological activity of extracellular HMGB1 [13]. The disulfide bond between Cys23 and Cys45 in the HMGB1 protein (disulfide HMGB1; oxidized HMGB1) causes inflammatory cytokine-stimulating activity [14]. In contrast, reduction in all cysteine residues (reduced HMGB1) results in chemotaxis-mediated activity [14]. When all cysteine residues are oxidized, HMGB1 is inactivated [15].

Moreover, redox modifications of HMGB1 play an important role in MSC-tumor cell communication [4]. Reduced HMGB1 promotes MSC chemotaxis and differentiation, while oxidized HMGB1 increases MSC stemness and proliferation [4]. MSCs treated with oxidized HMGB1 also increase cancer cell stemness and promote metastasis [4]. Thus, different forms of HMGB1 (oxidized or reduced) have different effects on MSCs and may have a different impact on cancer cells within TME; however, the mechanism is not completely elucidated.

Therefore, in this study, to elucidate the mechanism by which MSCs influence CRC growth promotion, we examined MT between MSCs and CRC cells and the effect of MT on CRC cells. We also examined the role of HMGB1 in MT and its redox modification.

## 2. Results

### 2.1. Effect of HMGB1 on MT

**Figure 1:**
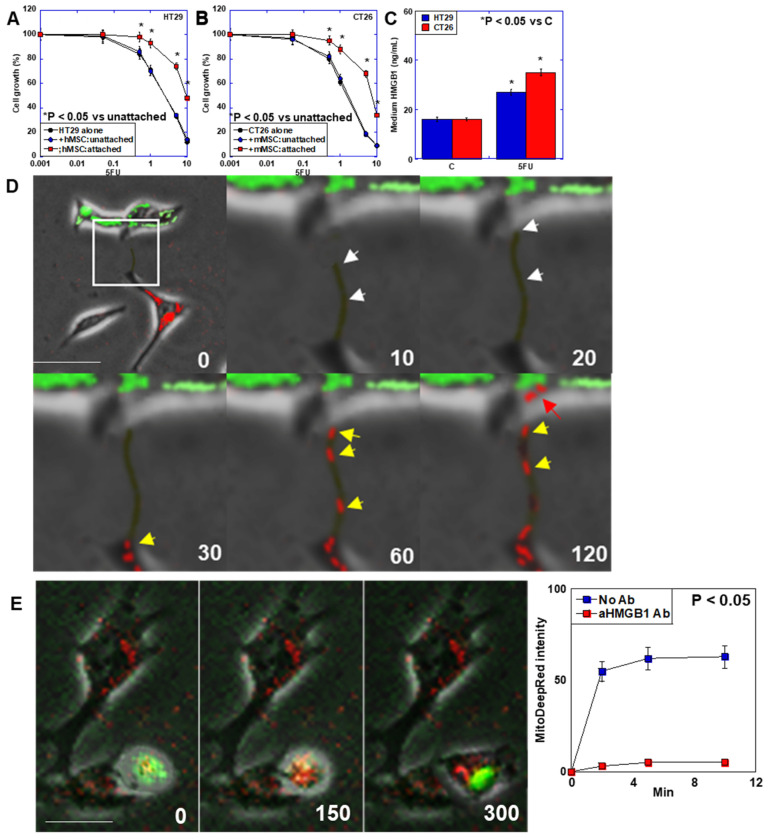
TNT formation and MT between MSCs and CRC cells. (**A**) Effect of coculture of HT29 and hBM-MSCs with attached or unattached conditions. (**B**) Effect of coculture of CT26 and mBM-MSCs with attached or unattached conditions. (**C**) HMGB1 concentration in medium of 5-FU-trated CRC cells. (**D**,**E**) MSCs were cocultured with 5-FU (1.5 μg/mL)-treated CRC cells with attached condition. Green color, CT26 cells; red color, mitochondria of BM-MSCs. (**D**) Timelapse analysis of TNT formation of mMSC to 5FU treated-mouse CT26 CRC cell. The 10 s to 120 s panels expand the range indicated by the white square in the 0 s panel. White arrow, TNT; yellow arrow, MSC’s mitochondria in TNT; red arrow, MSC’s mitochondria in CT2t cell. (**E**) Timelapse analysis of MT between hMSCs and HT29 human CRC cell treated with 5FU. Right panel, temporal change in HT29 cell number containing hMSC mitochondria; i.e., mitochondrial transferred HT29 cell number. Scale bar, 10 μm. Error bar, standard deviation from three independent trials. Statistical significance was calculated by an ordinary ANOVA test. TNT: tunneling nanotube; MT: mitochondrial transfer; CRC: colorectal cancer; hMSCs: human bone marrow-derived mesenchymal stem cells; mBM-MSCs: mouse bone marrow-derived mesenchymal stem cells; 5-FU: 5-fluorouracil.

Assessing the relationship between CRC cancer cells and bone marrow BM-MSCs, CRC and MSCs were cocultured in unattached or attached conditions (Figure 1A,B). In the unattached condition, HT29 and CT26 CRC cells showed cell growth at the same levels to those in non-cocultured cells, whereas both CRC cells showed enhanced cell growth in the attached condition. CRC cells treated with 5-FU increased the secretion of HMGB1 into the cultured medium (Figure 1C). To confirm MT from BM-MSCs to CRC cells, CRC cells (PKH67 labeled) and BM-MSCs (mitochondria labeled with Mito Deep red) were cocultured (Figure 1D,E). Under 5FU treatment, mBM-MSCs elongated TNT to the CT26 CRC cell. Mitochondria of mBM-MSCs were transferred to the CT26 cell through the TNT (Figure 1D). The mMSC TNT reached the CT26 cell at 20 s. The mMSC mitochondria entered into the TNT at 30 s. The mMSC mitochondria reached the CT26 cell cytoplasm. The hBM-MSCs attached to the HT29 CRC cell. Mitochondria of hBM-MSCs were transferred to the HT29 cell temporally (Figure 1E).

**Figure 2:**
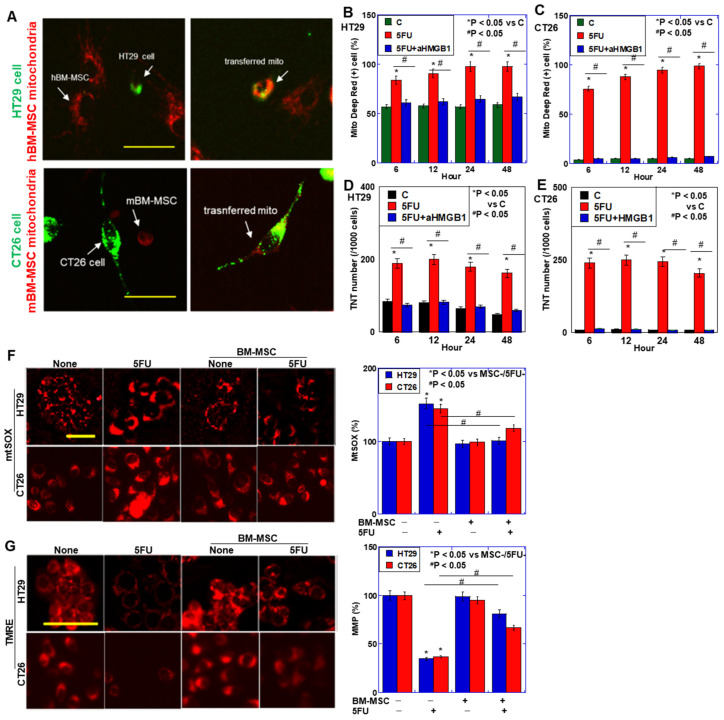
Effect of HMGB1 on mitochondrial transfer from BM-MSCs to CRC cells. (**A**) Fluorescence image of mitochondrial transfer from hBM-MSCs to HT29 cells and mBM-MSCs to CT26 cells. Scale bar, 50 μm. (**Left**) Mito Deep Red-labeled hBM-MSCs were cocultured with PKH67-labeled HT29 cells. (**Right**) hBM-MSC mitochondria were transferred from hBM-MSCs to PKH67-labeled HT29 cells. (**B**,**C**) The percentage of Mito Deep Red-positive CRC cells relative to all CRC cells. (**D**,**E**) TNT number in 1000 CRC cells. CRC cells were pretreated with 5-FU (1.5 μg/mL) or co-treated with 5-FU (1.5 μg/mL) and anti-HMGB1 antibody (αHMGB1, 10 μg/mL) for 48 h. CRC cells were cocultured with Mito Deep Red-labeled BM-MSCs for 6, 12, and 24 h. (**F**,**G**) Effects of coculture with BM-MSCs with 5-FU (1.5 μg/mL) and/or αHMGB1 (10 μg/mL) for 48 h on mitochondrial hydroxyradical (mtSOX) (**G**) and mitochondrial membrane potential (TMRE). Scale bar, 50 μm. Right panels, semi-quantification of fluorescent intensities of mtSOX and TMRE. Error bar, standard deviation from three independent trials. Statistical significance was calculated by an ordinary ANOVA test. hBM-MSCs: human bone marrow-derived mesenchymal stem cells; mBM-MSCs: mouse bone marrow-derived mesenchymal stem cells; HMGB1: high mobility group B-1; 5-FU: 5-fluorouracil; MMP: mitochondrial membrane potential; TNT: tunneling nanotube.

MCS mitochondria-transferred CRC cells were found in both CRC cells. MT from BM-MSCs to both CRC cells were significantly increased after coculture under 5-FU exposure, which was abrogated by anti-HMGB1 antibody treatment (Figure 2A–C). Similar to MT, TNT formation was also promoted by 5FU treatment, and the promoting effect of 5FU was suppressed by treatment with anti-HMGB1 antibody (Figure 2D,E). Thus, MT from MSCs to CRC cells occurred, which was enhanced by 5-FU. Since 5-FU increased HMGB1 secretion, HMGB1 might be associated with MT between CRC cells and MSCs.

Next, oxidative stress and mitochondrial membrane potential (TMRE) were examined (Figure 2F,G). Mitochondrial hydroxyradical (mtSOX) levels were increased by 5-FU treatment, which were abrogated by coculture with BM-MSCs. Mitochondrial membrane potentials (MMP) were decreased by 5-FU treatment, which were abrogated by coculture with BM-MSCs. In contrast, anti-HMGB1 antibody treatment diminished the effects of BM-MSCs on mtSOX and MMP.

### 2.2. Effect of CRC Cell Cultured Medium (CM) on BM-MSCs

**Figure 3:**
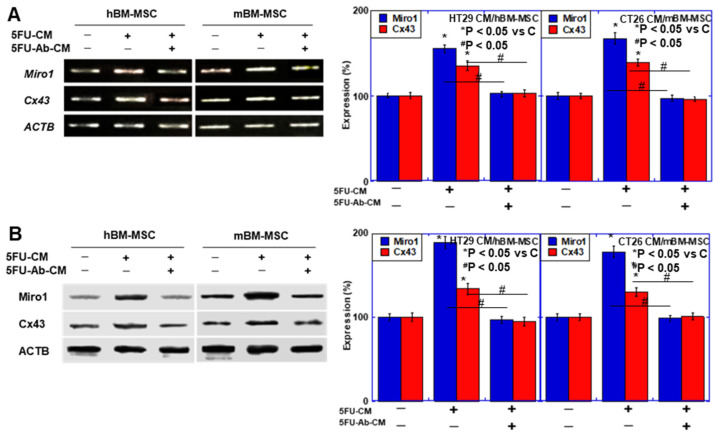
Effect of culture supernatant on mitochondrial-*transfer* associated factors in BM-MSCs. (**A**) Expression of genes associated with mitochondria transfer (*Miro1* and *Cx43*) in BM-MSCs. BM-MSCs were exposed to culture medium of CRC cells with or without αHMGB1 (10 μg/mL) for 48 h. CRC cells were pretreated with 5-FU (1.5 μg/mL). *ACTB* was used as a loading control. Right panels, semi-quantification of the signal densities in RT–PCR standardized with *ACTB* signal intensity. (**B**) Protein levels of Miro1 and Cx43 in BM-MSCs treated with same protocols. ACTB was used as a loading control. Right panels, semi-quantification of the signal densities in western blot standardized with ACTB signal intensity. Error bar, standard deviation from three independent trials. Statistical significance was calculated by an ordinary ANOVA test. hBM-MSCs: human bone marrow-derived mesenchymal stem cells; mBM-MSCs: mouse bone marrow-derived mesenchymal stem cells; 5-FU: 5-fluorouracil; CM: culture medium; HMGB1: high mobility group B-1; αHMGB1: anti-HMGB1 antibody; Miro1: mitochondrial Rho GTPase 1; Cx43: connexin43; ACTB: β-actin.

Mitochondrial Rho GTPase 1 (Miro1), attaching the mitochondria to the motor/adaptor complex [16] and connexin 43 (Cx43), a gap junction protein [17], are involved in TNT formation. Their expression is induced during MT, but their decrease suppresses MT [18]. We analyzed the effect of anti-HMGB1 antibody on expression of Miro1 and Cx43 in human BM-MSCs (hBM-MSCs) and mouse BM-MSCs (mBM-MSC) (Figure 3A,B). BM-MSCs exposed to CM of 5-FU-treated CRC cells increased mRNA expression of *Miro1* and *Cx43*, and protein levels of Miro1 and Cx43 compared to 5-FU untreated CM. In contrast, anti-HMGB1 antibody decreased mRNA expression of *Miro1* and *Cx43*, and protein levels of Miro1 and Cx43 compared to untreated CMs. This suggests that HMGB1 promotes the formation of TNT required for MT.

### 2.3. Effect of oxHMGB1 on MT

**Figure 4:**
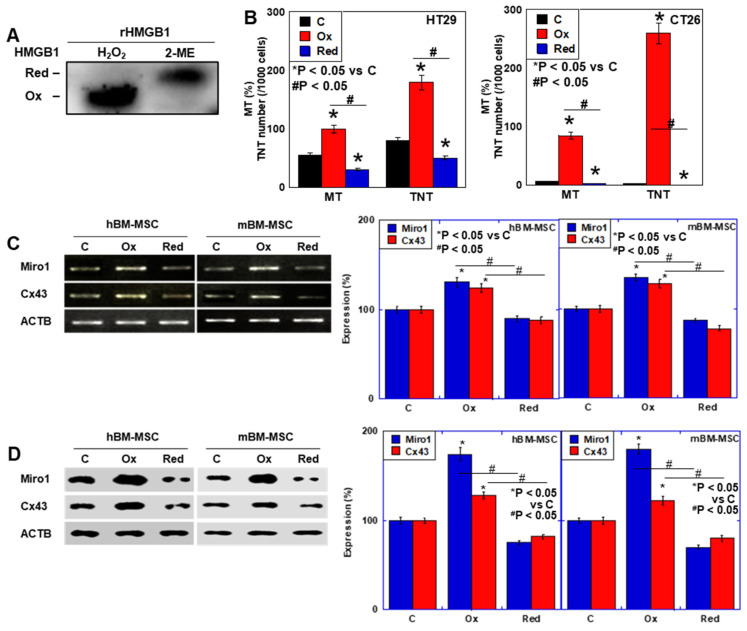
Effect of oxidized HMGB1 on mitochondrial transfer associated factors of BM-MSCs. (**A**) Western blotting of H_2_O_2_-treated rHMGB1 (oxHMGB1) and 2-merucaptoethanol-treated rHMGB1 (redHMGB1). (**B**) Effect of redox modification of HMGB1 (100 ng/mL) on MT and TNT formation in coculture of MSCs and CRC cells. (**C**) Expression of mitochondria transfer-associated genes (*Miro1* and *Cx43*) in BM-MSCs treated with or without oxHMGB1 (100 ng/mL) for 48 h. ACTB was used as a loading control. Right panels, semi-quantification of the signal intensities in RT–PCR standardized with *ACTB* signal intensity. (**D**) Protein levels of Miro1 and Cx43. ACTB was used as a loading control. Right panels, semi-quantification of the signal intensities in western blot standardized with ACTB signal intensity. Error bar, standard deviation from three independent trials. Statistical significance was calculated by an ordinary ANOVA test. MSC: mesenchymal stem cell; MT: mitochondrial transfer; TNT: tunneling nanotube; hBM-MSCs: human bone marrow-derived mesenchymal stem cells; mBM-MSCs: mouse bone marrow-derived mesenchymal stem cells; HMGB1: high mobility group B-1; rHMGB1: recombinant HMGB1; oxHMGB1: oxidized HMGB1; redHMGB1: reduced HMGB1; Miro1: mitochondrial Rho GTPase 1; Cx43: connexin43; ACTB: β-actin.

The above results suggest that HMGB1 promotes MT. To confirm this, we treated MSCs with recombinant HMGB1 (rHMGB1) and examined the expression of TNT formation-related factors (Figure 4). HMGB1 formed different protein construct by posttranscriptional modification, especially oxidation [18]. We then compared oxidized HMGB1 (oxHMGB1) and reduced HMGB1 (redHMGB1) on MT (Figure 3). The former is usually predominant in extracellular HMGB1 [19]. When rHMGB1 was treated with H_2_O_2_, all rHMGB1 was converted to oxHMGB1 (Figure 4A). In contrast, rHMGB1 treated with 2-mercaptoethanol turned all rHMGB1 into redHMGB1. We examined the effect of redox modification of HMGB1 on MT and TNT formation (Figure 4B). MT and TNT formation was promoted by oxHMGB1, but was inhibited by redHMGB1. We compared the effect of oxHMGB1 with redHMGB1 on mRNA and protein expression of Miro1 and Cx43 in BM-MSCs. oxHMGB1 significantly increased both *Miro1* and *Cx43* mRNA levels (Figure 4C). In contrast to oxHMGB1, redHMGB1 did not increase *Miro1* and *Cx43* mRNAs. oxHMGB1, but not redHMGB1, increased protein levels of Miro1 and Cx43 in BM-MSCs (Figure 4D). Thus, our results suggest that oxHMGB1, but not redHMGB1, promotes MT.

### 2.4. Effects of NF–κB Suppression on MT

**Figure 5:**
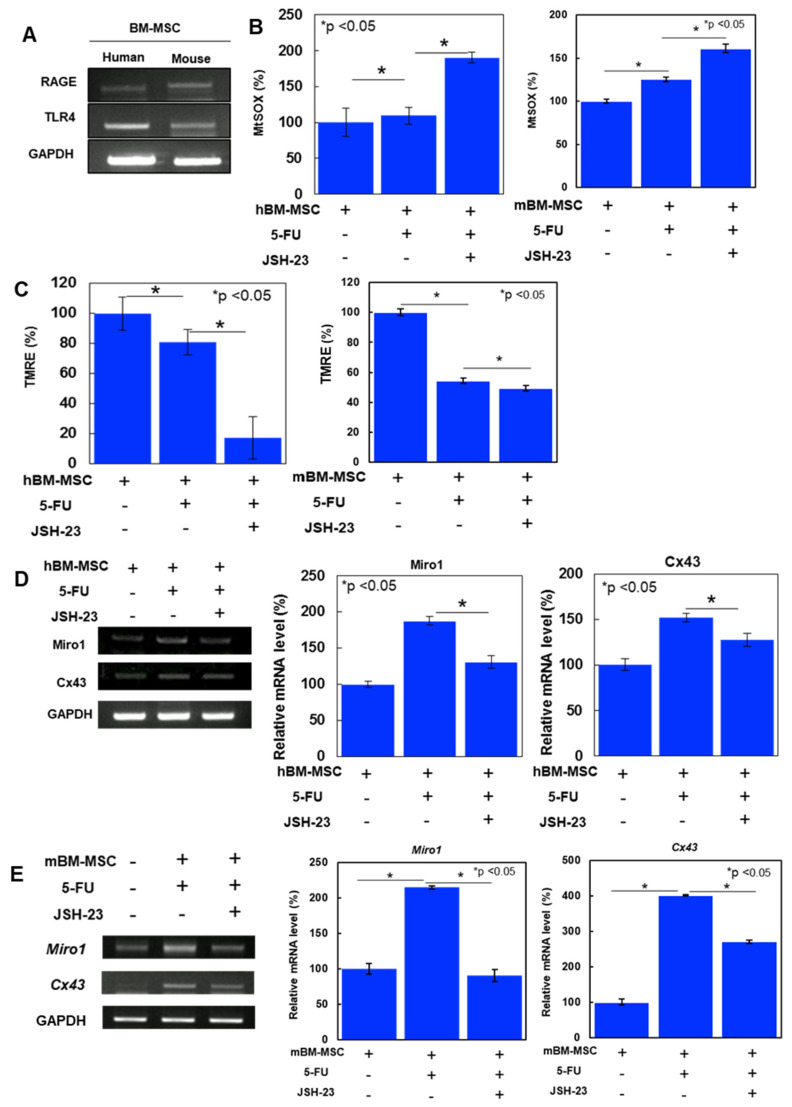
Effects of NF–κB inhibitor on mitochondrial function in CRC cells and mitochondrial transfer associated factors in BM-MSCs. (**A**) RT–PCR of RAGE and TLR4 as receptors for HMGB1 in BM-MSCs. (**B**,**C**) MtROS (**B**) and MMP (**C**) of CRC cells were examined. CRC cells were pretreated with or without 5-FU (1.5 μg/mL) for 48 h and cocultured with hBM-MSCs and then treated with or without a NF–κB inhibitor, JSH-23 (20 μM) for 24 h. (**D**,**E**) Expression of mitochondria transfer-associated genes (*Miro1* and *Cx43*) in BM-MSCs. hBM-MSCs (**D**) and mBM-MSCs (**E**). BM-MSCs were exposed to culture medium of 5-FU (1.5 μg/mL)-pretreated CRC cells and treated with or without JSH-23 (20 μM) for 48 h. GAPDH was used as a loading control. Right panels, Semi-quantification of the signal densities in RT–PCR standardized with *GAPDH* signal intensity. Error bar, standard deviation from three independent trials. Statistical significance was calculated by an ordinary ANOVA test. hBM-MSCs: human bone marrow-derived mesenchymal stem cells; mBM-MSCs: mouse bone marrow-derived mesenchymal stem cells; RAGE: receptor for advanced glycation end products; TLR4: toll-like receptor 4; mtROS: mitochondrial reactive oxidative species; mtSOX: mitochondrial hydroxyradical; MMP: mitochondrial membrane potential; TMRE: tetramethyl rhodamine; 5-FU: 5-fluorouracil; GAPDH: glyceraldehyde-3-phosphate dehydrogenase.

HMGB1 is known to activate nuclear factor NF–κB via RAGE and TLR4 [12,20]. Therefore, we evaluated the effect of NF–κB on MT in 5-FU-treated CRC cells (Figure 5). First, we examined the mRNA expression of RAGE and TLR4 in BM-MSCs (Figure 4A). Human and mouse BM-MSCs expressed RAGE and TLR4. Next, we examined the effect of cocultured BM-MSCs on MtSOX and MMP in HT29 and CT26 cells (Figure 5B,C). When cocultured cells were treated with JSH-23, an NF–κB inhibitor, 5-FU induced mitochondrial reactive oxygen species (ROS) increase and MMP decrease became further pronounced to 5-FU alone. Next, we examined the involvement of NF–κB in the mRNA expression of *Miro1* and *Cx43* in BM-MSCs (Figure 5D). BM-MSCs exposed to CM from 5-FU treated CRC cells increased the expression of *Miro1* and *Cx43*, which was suppressed by JSH-23. Thus, our results suggest that NF–κB activation is required for induction of MT by HMGB1.

### 2.5. Significance of MT in CRC Cells

**Figure 6:**
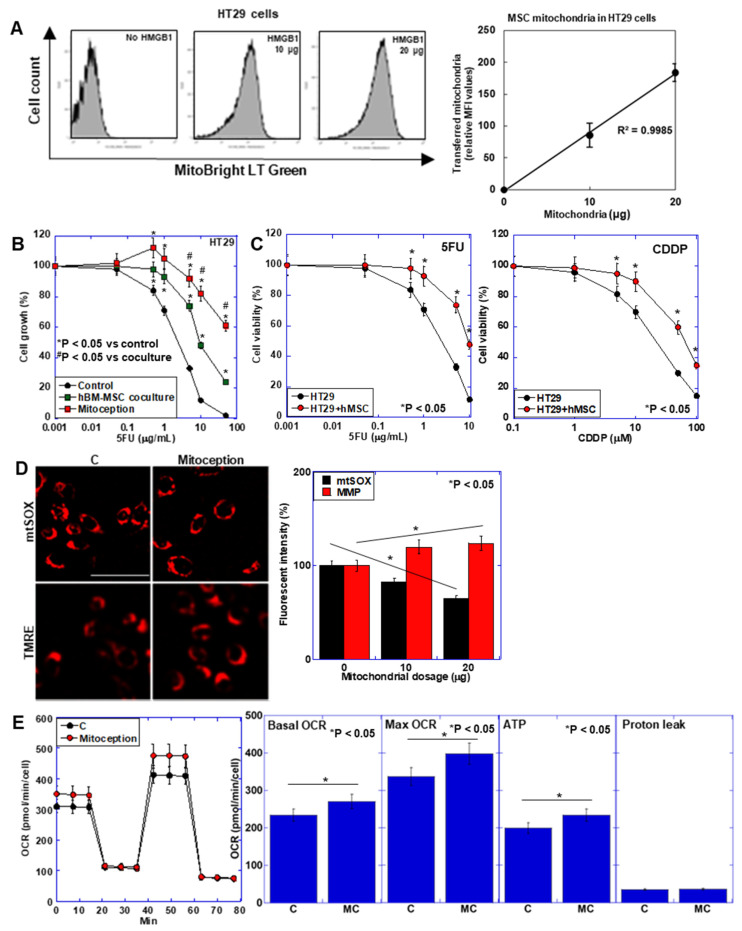
Effect of artificial mitochondria transfer (mitoception) on metabolism of CRC cells. (**A**) FACS analysis of HT29 cells mitocepted with increasing amounts of hBM-MSCs-derived mitochondria. hBM-MSCs mitochondria were labeled with MitoBright LT Green. Right panel, relationship between loaded mitochondrial amounts and transferred mitochondrial fluorescent intensities. (**B**) Effect of hBM-MSCs coculture or mitoception on 5-FU sensitivity of HT29 cells. (**C**) Sensitivities to 5FU and CDDP of HT29 cells cocultured with hMSCs. Cell viability was determined by counting the number of cells after treatment with different concentrations of 5-FU or CDDP for 48 h (**B**,**C**). Insert, MT-positive cell (%). (**D**) Effect of mitoception on mtROS and MMP. Right panel, semi-quantification of fluorescent intensities. (**E**) Effect of mitoception on OXPHOS. Error bar, standard deviation from three independent trials. Statistical significance was calculated by an ordinary ANOVA test. FACS: fluorescence-activated cell sorting; MFI: mean fluorescence intensity; hBM-MSCs: human bone marrow-derived mesenchymal stem cells; 5-FU: 5-fluorouracil; ROS: reactive oxidative species; mtSOX: mitochondrial hydroxyradical; MMP: mitochondrial membrane potential; TMRE: tetramethyl rhodamine; OXPHOS: oxidative stress; OCR: oxygen consumption rate; Max: maximum; CDDP: cisplatin; MT: mitotransfer; hMSC: human bone marrow mesenchymal stem cells.

We next investigated the effect of MT from BM-MSCs on CRC cells (Figure 6). Using the mitoception method [21], mitochondria extracted from hBM-MSCs were artificially introduced into 5-FU-treated HT29 cells (Figure 6A). By mitoception, BM-MSC-mitochondria were transferred to HT29 cells in a dose-dependent manner. When we examined 5-FU sensitivity, HT29 cells that transferred hBM-MSC mitochondria showed enhanced 5-FU resistance, as well as cocultured HT29 cells with hBM-MSC compared to control cells (Figure 6B). Sensitivities to 5-FU and cisplatinum (CDDP) of hBM-MSCs-cocultured HT29 cells were also decreased (Figure 6C). Mitoception decreased mitochondrial ROS and increased MMP compared to those in control cells in a dose-dependent manner (Figure 6D). Oxidative phosphorylation was also enhanced by mitoception (Figure 6E).

**Figure 7:**
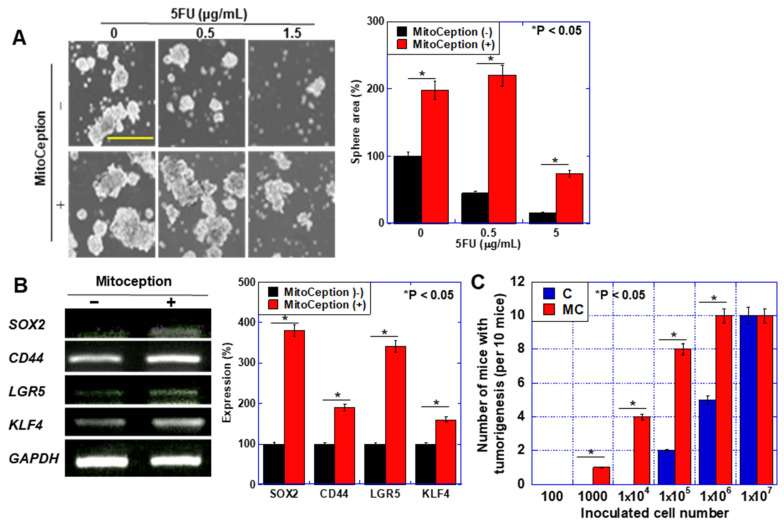
Effect of artificial mitochondria transfer (mitoception) on stemness of CRC cells. (**A**) Effect of mitoception on sphere formation in HT29 cells. Sphere formation was examined in 10,000 cells for 7 days. Pictures were images of phase-contrast microscopy. Scale bar, 300 μm. Right panel, number of spheres was counted with microscopy at 7 days. (**B**) Expression of stemness-associated genes (*Sox2*, *CD44*, *LGR5*, and *KLF4*). *GAPDH* expression was used as a loading control. Right panel, semi-quantification of the signal densities in RT–PCR standardized with *GAPDH* signal intensity. (**C**) Tumorigenicity of HT29 cells with mitoception. Cells were inoculated subcutaneously in mice. Error bar, standard deviation from three independent trials. Statistical differences were calculated by an ordinary ANOVA test with Bonferroni correlation from five mice. hBM-MSCs: human bone marrow-derived mesenchymal stem cells; 5-FU: 5-fluorouracil; GAPDH: glyceraldehyde-3-phosphate dehydrogenase, SOX2: sex determining region Y box 2; LGR5: leucine-rich repeat-containing G-protein coupled receptor 5; KLF4: Krüppel-like factor 4.

Since stemness has been emphasized as a cause of chemotherapy resistance [10], we investigated the effect of MT on stemness (Figure 7). hBM-MSC mitochondria-transferred HT29 cells showed enhanced sphere formation, even under 5-FU (Figure 7A). When mRNA expression of stemness-associated genes in mitochondria-transferred HT29 was examined, expression of *SRY-box transcription factor 2* (*Sox2*), *CD44*, *leucine-rich repeat-containing G-protein coupled receptor 5* (*LGR5*), and *Krüppel-like factor 4* (*KLF4*) were all increased (Figure 7B). Untreated and mitocepted HT29 cells were inoculated subcutaneously into the back of nude mice (10 mice each) (Figure 7C). There was no difference between control cells and mitocepted cells at inoculation with 1 × 10^7^ cells. In contrast, mitocepted cells showed higher tumorigenesis when inoculated with 1 × 10^3^ to 1 × 10^6^ cells compared to control cells. Thus, cancer cells transfected with BM-MSC mitochondria showed enhanced stemness and increased chemotherapy resistance.

### 2.6. Effects of MT Inhibition in a Mouse Subcutaneous Tumor Model

**Figure 8:**
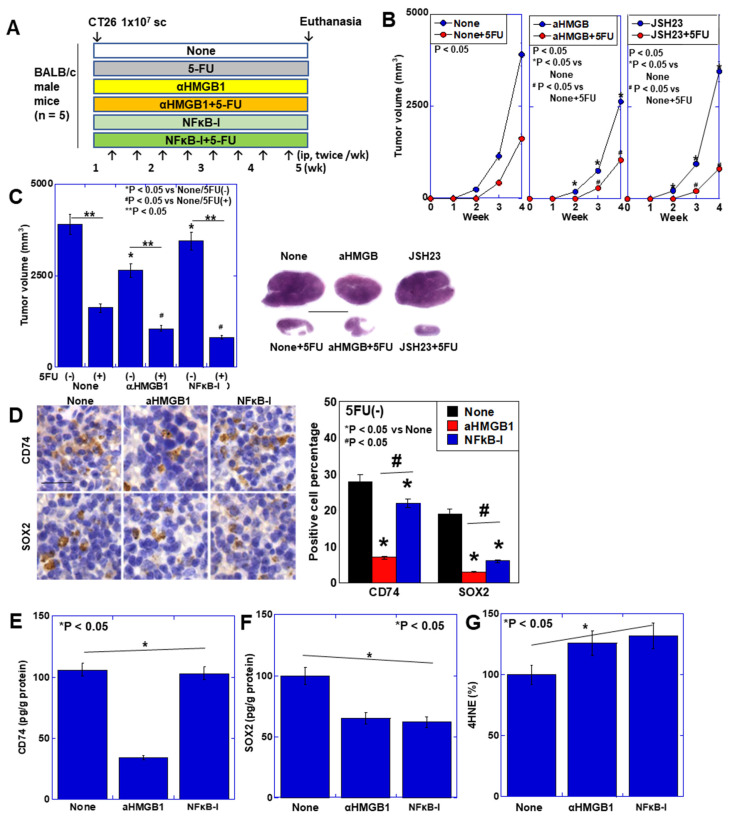
Effect of inhibition of MT in mouse subcutaneous tumor model. (**A**) Experimental protocol. 5-FU (30 mg/kg BW), and αHMGB1 (5 μg/mouse) [22] or NF–κB-I (JSH-23, 20 mg/kg BW) [23] were administered intraperitoneally twice a week. (**B**,**C**) Effect of MT inhibition on tumor growth; time course (**B**) and tumor volume at 4 weeks after inoculation (**C**) Right panel, loupe images of the maximum cut surface of representative tumors stained with hematoxylin and eosin. Scale bar, 1 cm. (**D**) Immunohistochemistry of CD73 and SOX2 in tumors. Scale bar, 50 μm. (**E**,**F**) Contents of mBM-MSC-related proteins, CD73 (**E**) and SOX (**F**) in tumor tissues. (**G**) Contents of ROS levels (4HNE). s. Error bar, standard deviation from five mice. Statistical differences were calculated by an ordinary ANOVA test with Bonferroni correlation. Miro1: mitochondrial Rho GTPase 1; SOX2: sex determining region Y box 2; MT: mitochondrial transfer; 4HNE: 4-hydroxynonenal; αHMGB1: anti-high mobility group box-1 antibody; 5-FU: 5-fluorouracil; NF–κB-I: nuclear factor–κB inhibitor, JSH-23.

To examine the in vivo effects of MT, CT26 cells were inoculated subcutaneously in syngeneic BALB/c mice and treated with a HMGB1 neutralizing antibody or NF–κB inhibitor (JSH-23) with or without 5-FU (Figure 8A). Tumor growth in time course (Figure 8B) and tumor volume at 4 weeks after inoculation (Figure 8C) showed that MT inhibition suppressed tumor growth, especially HMGB1 antibody treatment. The tumor suppressive effect of 5-FU was enhanced by MT inhibition. For evaluating MSC migration into the tumors, MSC marker CD74- or sex determining region Y box 2 (SOX2)-positive cells in tumor tissues were detected by immunohistochemistry (Figure 8D). The anti-HMGB1 antibody strongly inhibited the infiltration of CD74- or SOX2-positive MSCs into tumors, whereas the NFκB inhibitor had a weak effect. For confirmation, MSC-associated protein levels of CD74 and SOX2 in tumor tissues were examined. CD74 was reduced only with HMGB1 antibody treatment (Figure 8E). A stemness marker SOX2 protein decreased by both anti-HMGB1 antibody and NFκB inhibitor (Figure 8F). ROS levels were increased by MT inhibition (Figure 8G). Thus, MT inhibition suppressed stemness and increased 5-FU sensitivity.

### 2.7. Effect of In Vivo MT on CRC Cells in Miro1-Knockdown Mice

**Figure 9:**
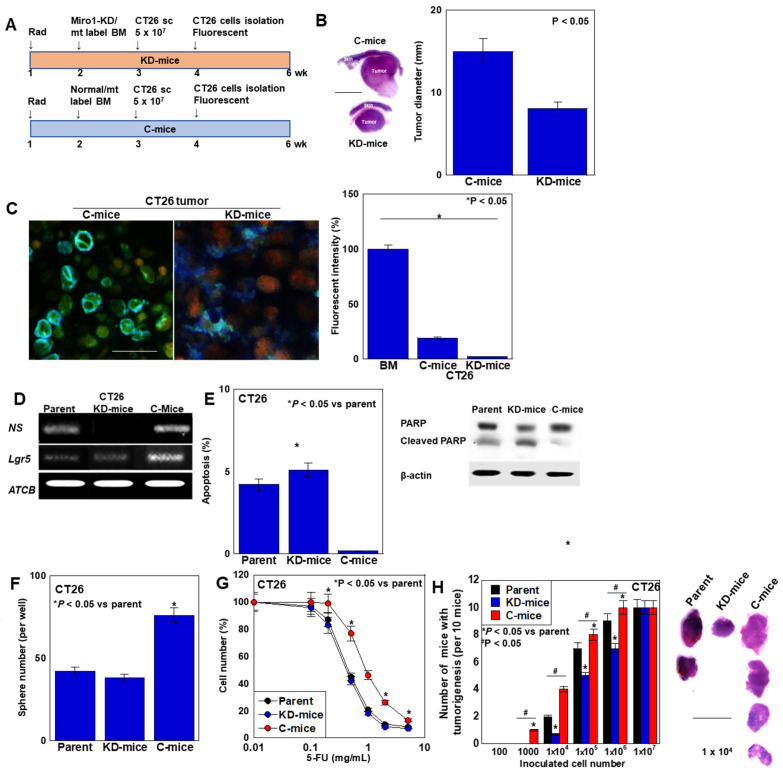
Effect of in vivo MT on CT26 cells in mice with *Miro1*-knockdown. (**A**) Experimental protocol. BALB/c mice were transplanted with *Miro1*-KD and mitochondria labeled BM cells (KD-mice) or mitochondria labeled BM cells (C-mice). CT26 cells were inoculated subcutaneously into KD- or C-mice. From the tumors, CT26 cells were isolated at 4 weeks after inoculation. (**B**) Effect of *Miro1*-KD on tumor growth. Left panel, loupe images of the maximum cut surface of representative tumors in each group (hematoxylin and eosin staining). Scale bar, 1 cm. (**C**) Fluorescent intensity in transplanted BM cells, CT26 cells isolated from KD- or c-mice in comparison with those in parental CT26 cells. Left panels, representative fluorescence images of CT26 tumors. Scale bar, 50 μm. (**D**–**H**) Effect of *Miro1*-KD on stem cell phenotypes in CT26 cells isolated from KD- or c-mice. Expression of stemness-associated genes, *NS* and *Lgr5* (**D**), apoptosis; Right panel, PARP cleavage by western blot analysis, (**E**), sphere formation (**F**), 5-FU sensitivity (**G**), and tumorigenicity (**H**). Right panel, loupe images of the maximum cut surface of representative tumors in 1 × 10^4^-inoculated group stained with hematoxylin and eosin. Scale bar, 1 cm. Error bar, standard deviation from five mice. Statistical differences were calculated by an ordinary ANOVA test with Bonferroni correlation. BM: bone marrow; C-mice: mice whose BM replaced with mitochondria labeled BM cells; KD-mice: mice whose BM replaced with *Miro1*-KD and mitochondria labeled BM cells; MSC: mesenchymal stem cells; Miro1: mitochondrial Rho GTPase 1; NS: nucleostemin; Lgr5: leucine-rich repeat-containing G-protein coupled receptor 5; KD: knockdown by siRNA; parent: parental CT26 cells without inoculation.

To examine the effect of in vivo MT on CRC cells, CT26 cells were inoculated subcutaneously into mice transplanted with *Miro1*-knockdown (KD) and mitochondria-labeled bone marrow (BM)(KD-mice) or mice transplanted with mitochondria-labeled BM (C-mice). CT26 cells isolated from subcutaneous tumors were cultured in regular medium (Figure 9A). Tumor size was decreased by *Miro1* KD (KD-mice) (Figure 9B). Next, mitochondrial fluorescence was compared between CT26 cells from KD-mice and CT26 cells from C-mice (Figure 9C, left panels). In semi-quantitated fluorescence intensities with reference of mitochondria-labeled BM cells, CT26 cells from C-mice showed 15% intensity of labeled BM, whereas CT26 cells from KD-mice showed only 1%, which suggested *Miro1*-KD reduced in vivo MT. CT26 cells from tumors in C-mice showed increased expression of *nucleostemin* and *Lgr5* in comparison with parent CT26 cells and CT26 cells from tumors in KD-mice (Figure 9D). CT26 cells from tumors in C-mice showed decreased apoptosis (Figure 9F), increased sphere forming ability (Figure 9F), 5-FU resistance (Figure 9G), and enhanced tumorigenicity (Figure 9H). However, these alterations were abrogated in CT26 cells from tumors in KD-mice. Thus, in vivo MT from host BM-MSCs to cancer cells was suggested to increase cancer cell stemness.

## 3. Discussion

In the present study, we elucidated a novel mechanism that MT from BM-MSC is responsible for enhanced stemness in CRC cells. HMGB1 released from CRC cells in response to chemotherapy-induced damage promotes MT of BM-MSCs to CRC cells, resulting in enhanced stemness and anticancer drug resistance in CRC cells. Furthermore, among the HMGB1 redox modifications, we revealed that oxHMGB1, but not redHMGB1, provides an intracellular signal via NF–κB in BM-MSCs that triggers MT from BM-MSCs.

In this study, mechanical introduction of mitochondria into CRC cells by mitoception resulted in enhanced sphere formation and increased expression of stem cell markers and resistance to 5-FU, which are associated with enhanced stemness in CRC cells. The results suggest that extrinsic mitochondria might enhance cancer stemness. Energy metabolism in cancer stem cells is dependent on oxidative phosphorylation (OXPHOS) in comparison with differentiated cancer cells [24]. In this study, it appears that oxidative phosphorylation, ROS generation, and energy production were normalized in cancer cells that received normal mitochondria from MSCs. Mitochondria are not only powerhouses in cancer cells, but also regulators of cell survival, cell death, proliferation, motility, and stemness [25,26]. Mitochondrial biogenesis enhances the expression of markers for sphere formation, stemness, pluripotency, and epithelial–mesenchymal transition [27]. Furthermore, promotion of mitochondrial biogenesis and OXPHOS are associated with increased stemness at transition from a primed state to a naive state of embryonal stem cells [28]. However, the detailed mechanisms by which mitochondria enhance stemness remain unclear.

In this study, we used BM-MSCs as mitochondrial donors in MT. Stem cells maintain their lineage through asymmetric cell division. Interestingly, mitochondria are also distributed asymmetrically during cell division [29,30]; new mitochondria are selectively distributed in stem cell lineage, which have low OXPHOS activity. In contrast, old mitochondria are distributed in differentiating cells, and these have high OXPHOS activity. Umbilical cord MSCs exhibit energy metabolism via OXPHOS, but high stemness correlates with relatively low OXPHOS activity [31]. In our study, the increase in OXPHOS in mitocepted CRC cells was at low levels. MSCs as mitochondrial donors endow cancer cells with stemness by transferring their own mitochondria associated with high stemness to cancer cells. Since MT enhances stemness, MT may enhance not only chemotherapy resistance but also cancer metastasis [32].

Damage to mitochondria by anticancer drugs induces cell death, but at the same time induces adaptation of cancer cells to the anticancer drugs in terms of energy metabolism and redox balance, thereby inducing drug resistance [33]. Our data suggest that anticancer drug-induced MT enhances anticancer drug resistance through promotion of stemness and suppression of ROS production in cancer cells. Along with MT-induced stemness enhancement, mitochondrial ROS reduction due to introduction of normal mitochondria by MT is thought to be important in the acquisition of anticancer drug resistance [34]. Our data also show that mitochondrial transfer normalizes MMP in CRC cells and suppresses the induction of excessive ROS production by 5-FU. In Jurkat cells, injured mitochondria by chemotherapy are transferred to MSCs, thereby decreasing intracellular ROS and acquiring anticancer drug resistance [9]. In our results, cancer cells are supplied with normal mitochondria from BM-MSCs rather than excreting the impaired mitochondria into MSCs. However, both of these processes reduce ROS in cancer cells, leading to resistance to anticancer drugs.

HMGB1 is known as a migration factor for BM-MSCs [35]. oxHMGB1 plays roles in migration and retaining stemness of BM-MSCs [4]. BM-MSCs that have migrated into cancer tissues promote the stemness of cancer cells and enhance their metastatic potential [4]. Interestingly, this effect requires cell-to-cell adhesion [4]. In our data, attached coculture, but not unattached coculture, increased cell growth.

In contrast to oxHMGB1, redHMGB1 confers stronger migration ability to MSCs while promoting their differentiation into bone and endothelial cells [36,37]. This difference in the action of oxHMGB1 and redHMGB1 on cancer cells may be attributed to their differential action in MT. High levels of ROS are known to oxidize HMGB1 and release oxHMGB1 to the outside of cells [38]. It is well known that anticancer drugs induce intracellular ROS, which injure DNA to provide cell death [39]. This suggests that oxHMGB1 is released from cancer cells by anticancer drugs [40]. In other words, chemotherapy may promote the release of oxHMGB1 from cancer cells and induce MT from BM-MSCs.

In this study, we focused on Cx43 and Miro1 as factors responsible for MT in BM-MSCs. MT can be achieved by endocytosis [41], macropinocytosis [42], or TNTs containing a kinesin translocation system [43]. Cx43 and Miro1 are proteins that control TNT formation [44,45]. In a mouse model of acute lung injury, BM-MSCs regulate TNT formation and transferring mitochondria to alveolar cells via Cx43 [46]. Overexpression of Miro1 significantly improves MT efficiency in induced-pluripotent-stem-cell-derived-MSCs [47]. We showed that NF–κB induces *Miro1* gene expression. NF–κB is located downstream of RAGE and TLR4 signals, which are activated by HMGB1 to induce inflammatory responses [48]. HMGB1 is known to be released from necrotic cells [11], and HMGB1 is also a marker of tissue damage [49]. These are thought to be mechanisms by which tissue damage markers activate MSCs and promote tissue protection and regeneration. This mechanism represents feedback in normal tissues and is linked to cancer cell viability and treatment resistance.

Our results suggest that NF–κB plays an important role in promoting MT. NF–κB is activated by various intracellular signals including RAGE. RAGE expression was confirmed in the MSCs we used. The redHMGB1–RAGE axis induces MSC differentiation [50], whereas AGE, also a RAGE ligand, suppresses differentiation [51]. We have also reported that HMGB1 and AGE express different effects through RAGE [52]. Furthermore, redHMGB1, oxHMGB1, and glycated HMGB1 have different activation effects on RAGE and provide different phenotypes [4]. Nuclear translocation of the NF–κB p65 is also at high levels in oxHMGB1 and at low levels in redHMGB1 [14]. Regarding the diversity of intracellular signals in RAGE, complex formation between RAGE, one of G protein-coupled receptors (GPCRs), and other GPCRs such as the angiotensin receptor (AT1) has been pointed out [53]. When RAGE forms a complex with AT1, it activates Gi and NF–κB. This suggests that the diversity of G protein subunits that mediate RAGE signals results in the diversity of RAGE signals.

In our in vitro studies, hBM-MSCs were cultured in DMEM, mBM-MSCs in stem cell medium, and DMEM was used for coculture with CRC cells. This may lead to changes in phenotypes in mouse MSCs. In fact, TNT formation is affected by various factors such as the cellular environment and stress conditions [54]. In addition, MTs change depending on the health of MSCs [55], and the culture environment can regulate the mitochondrial transplantation ability [56]. For this reason, MSCs cultured in DMEM may not show their original stemness. It is important to consider the extent to which BM-MSCs maintain stemness after leaving the bone marrow niche and migrating into cancer tissues or peripheral tissues. Because the oxHMGB1 we examined in this study has the effect of maintaining the stem cell stemness of BM-MSCs [4], it is possible that oxHMGB1 confers resistance to environmental changes on MSCs and helps maintain the activity of MSCs in non-bone marrow tissues.

When BM-MSCs were cocultured with CRC cells damaged by 5-FU, MT from BM-MSCs was promoted by oxHMGB1 released from cancer cells. As a result, CRC cells acquired stemness and drug resistance. These results suggest that targeting oxHMGB1 may serve as a new therapeutic strategy to suppress the malignant phenotypes of CRCs and increase therapeutic efficacy.

## 4. Materials and Methods

### 4.1. Cell Lines and Reagents

HT29 human carcinoma cell line was purchased from Dainihon Pharmacy Co. (Tokyo, Japan). CT26 murine colon carcinoma cell line was gifted by Professor I. J. Fidler (MD Anderson Cancer Center, Houston, TX, USA). UE7T-13 human bone marrow-derived MSC line (hBM-MSC) was obtained from the Health Science Research Resources Bank (JCRB1154; Japan Health Sciences Foundation, Tokyo, Japan). Cells were cultured in Dulbecco’s modified Eagle’s medium supplemented with 10% fetal bovine serum (FBS, Sigma Chemical Co., St. Louis, MO, USA) at 37 °C in 5% CO_2_. HT29 cells and CT26 cells were surface-labeled with PKH67 (Sigma) according to the manufacturer’s instructions.

In coculture of CRC cells with BM-MSCs (Figure 1), UniWells (Fuji Film, Osaka, Japan) was used for unattached coculture; CytoSelect (Cosmo-Bio, Tokyo, Japan) was used for attached coculture. In both coculture systems, CRC cells (5 × 10^3^) and BM-MSCs (5 × 10^3^) were seeded on culture wells according to the manufacturer’s instructions.

For oxidization of HMGB1, recombinant human HMGB1 (Biolegend, San Diego, CA, USA) was incubated with 50 μM H_2_O_2_ (Wako) on ice for 1 h. For reduction in HMGB1, recombinant human HMGB1 (Biolegend) was incubated with 50 mM 2-mercaptoethanol (Sigma) on ice for 1 h.

HT29 and CT26 cells were treated with 5-fluorouracil (5-FU, 1.5 μg/mL and 0.5 μg/mL for 48 h, Sigma) or co-treated with 5-FU (1.5 μg/mL and 0.5 μg/mL for 48 h, Sigma) and antihuman HMGB1 antibody (10 μg/mL for 48 h, Biolegend). MSCs were treated with oxHMGB1 (100 μg/mL for 48 h, Biolegend) or the specific NF–*κ*B inhibitor JSH-23 (20 μM for 48 h) (MedChemExpress, Monmouth Junction, NJ, USA).

### 4.2. Mouse BM-MSC (mBM-MSC) Preparation

BALB/c mice (5-week old, male, SLC Japan, Shizuoka, Japan) were euthanized by cervical dislocation under anesthesia, and bone marrow cells were harvested by flushing the bone marrow from the femur with regular DMEM (WAKO). After centrifugation at 1500 rpm for 5 min, the pellet was suspended in PBS (WAKO). Red blood cells were lysed with RBC lysis buffer (PluriSerect Life Science, Leipzig, Germany), followed by gentle vortexing at room temperature. Centrifugation was repeated at 1500 rpm for 5 min, and the pellet was resuspended and cultured in MSC culture medium (MesenCult-ACF Plus, Veritas, Tokyo, Japan) for 3 days. Floating cells were carefully removed by PBS washing.

### 4.3. Coculture of CRC Cells with BM-MSCs

PKH67-labeled HT29 cells and CT26 cells (5 × 10^5^ cells/well) were cultured in 6 wells for 48 h with 5-FU (1.5 μg/mL and 0.5 μg/mL, Sigma) or cotreated with 5-FU (1.5 μg/mL and 0.5 μg/mL, Sigma) and anti-HMGB1 antibody (10 μg/mL for 48 h, Biolegend). Then, cells were cocultured with half the number of Mito Deep Red-labeled BM-MSCs. The images were captured with a BZ-X710 All-in-One fluorescence microscope (KEYENCE, Osaka, Japan) and analyzed on a computer. The fluorescence intensity was measured using NIH ImageJ software (version 1.52, Bethesda, MD, USA).

### 4.4. Cell Count

To count MT and TNT, 20 microscopic fields by 40× magnification and 50 cells in each field were screened. The captured images were analyzed on a computer, and the number of cells was counted using ImageJ software (version 1.52; NIH, Bethesda, MD, USA).

### 4.5. Mitoception

Mitochondria were isolated from hBM-MSCs using the Mitochondria Isolation Kit for Tissue (ThermoFisher Scientific, Waltham, MA, USA), according to the manufacturer’s instructions. To obtain a mitochondria pellet with low contamination from other compounds, centrifugation was performed at 3000× *g* for 15 min. Typical mitochondria yield is 100 μg per million BM-MSCs. The mitochondria preparation was resuspended in DMEM on ice and used immediately for the artificial transfer. HT29 cells were seeded 5 × 10^5^ cells per 6-well dish and treated with or without 5-FU (1.5 μg/mL for 24 h, Sigma) the day before the artificial transfer. Cells were counted and the additional amounts of mitochondria were adjusted accordingly. The amounts show hBM-MSC mitochondria (μg of proteins) for 1 × 10^5^ HT29 cells. The mitochondria suspension was added slowly and close to the bottom of the well. The well was centrifuged at 1500× *g* for 15 min at 4 °C. The following day, cells were washed with PBS and analyzed.

### 4.6. Sphere Assay

CRC cells (1 × 10^6^) were cultured with 5-FU (1.5 μg/mL for 48 h, Sigma) and mitocepted with hBM-MSCs mitochondria. The following day, cells were seeded onto uncoated bacteriological 35 mm dishes (Coning Inc., Corning, NY, USA) in 3D Tumorsphere Medium XF (Sigma). After 7 days of culture, images of the spheres were acquired using a BZ-X710 All-in-One fluorescence microscope (KEYENCE, Osaka, Japan).

### 4.7. Mitochondrial Imaging

Mitochondrial functions were examined using fluorescent probes. Cells were incubated with the probes for 30 min at 37 °C and then imaged using a BZ-X710 All-in-One fluorescence microscope (KEYENCE, Osaka, Japan). We used MitoROS (superoxide, 10 μM, AAT Bioquest Inc., Sunnyvale, CA, USA) to assess oxidative stress, MitoTrackerTM Deep Red FM (Thermo Fisher Scientific, Tokyo, Japan) and MitoBright LT Green (Dojindo, Kumamoto, Japan) to assess mitochondrial volume, and tetramethylrhodamine ethyl ester (TMRE) (200 nM, Sigma) to assess mitochondrial membrane potential. The captured images were analyzed on a computer, and the fluorescence intensity was measured using NIH ImageJ software (version 1.52, Bethesda, MD, USA).

### 4.8. Reverse Transcription–PCR (RT–PCR)

To assess mRNA expression, RT–PCR was performed with 2 µg total RNA extracted from cells using TRI REAGENT (Molecular Research Center, Inc., Cincinnati, OH, USA) according to the manufacturer’s protocol. cDNA was synthesized with 0.5 µg total RNA using a High Capacity cDNA Reverse Transcription Kit (Applied Biosystems, Waltham, MA, USA). The primer sets are listed in Table 1 and were synthesized by Sigma Genosys (Ishikari, Japan). PCR products were electrophoresed on a 2% agarose gel and stained with ethidium bromide. *β-actin*, *GAPDH* and *α-tubulin* mRNA was also amplified for use as an internal control. Images were captured on a computer and the signal strength was measured using NIH ImageJ software (version 1.52, Bethesda, MD, USA).

### 4.9. Protein Extraction

To prepare whole cell lysates, cells were washed twice with cold PBS, harvested, and lysed with RIPA buffer containing 0.1% sodium dodecyl sulfate (SDS) (Thermo Fisher) [57]. Cell fractions were extracted by processing the cells with a Cell Fractionation Kit (Abcam, Cambridge, UK), according to the manufacturer’s instructions. Protein assays were performed using a Protein Assay Rapid Kit (Wako, Osaka, Japan).

### 4.10. Immunoblot Analysis

Whole cell lysates were prepared as previously described [57]. Lysates (50 μg) were subjected to immunoblot analysis using 12.5% SDS–polyacrylamide gels, followed by electrotransfer onto nitrocellulose membranes (Bio-Rad, Hercules, CA, USA). The membranes were incubated with primary antibodies and then with peroxidase-conjugated IgG secondary antibodies (MBL, Nagoya, Japan). Antibodies against Miro1 (Abcam, Cambridge, UK), and HMGB1 (Abcam, Cambridge, UK) were used for primary antibodies. Antibodies against β-actin (Oncogene Research Products, Cambridge, MA, USA) and GAPDH (Proteintech, Tokyo, Japan) were used to assess protein loading. Binding of the immune complex was visualized using a CSA system (DAKO, Carpinteria, CA, USA). Images were captured on a computer and the signal strength was measured using NIH ImageJ software.

### 4.11. Flow Cytometry

Fluorescence Activated Cell Sorting (FACS) experiments were performed using a Moxi GO II (AS ONE, Osaka, Japan). Data are expressed with the mean fluorescence intensity (MFI) values.

### 4.12. Animal Model

BALB/c mice (4-week old, male) were purchased from SLC. The animals were maintained in a pathogen-free animal facility at 23 °C and 50% humidity, under a 12 h light/dark cycle. The animal study was conducted in accordance with the institutional guidelines approved by the Committee for Animal Experimentation of Nara Medical University, Kashihara, Japan, following current regulations and standards of the Japanese Ministry of Health, Labor and Welfare (approval nos. 11528, 11569, 11596, 11725, 11716, 12777).

### 4.13. Subcutaneous Tumor Model

CT26 cells (1 × 10^7^ per mouse) were inoculated into the scapular subcutaneous tissues of 30 BALB/c mice (5-week old, male). They were divided to 6 groups each 5 mice; group 1, no treatment (None); group 2, 5-FU (10 mg/kg BW ip, Sigma); group 3, anti-HMGB1 antibody (0.5 mg/kg BW ip, Biolegend); group 4, anti-HMGB1 antibody + 5-FU; group 5, NFκB inhibitor (JSH-23, 20 mg/kg BW ip, MedChemExpress); group 6, NFκB inhibitor + 5-FU. In each group, drugs were administered twice a week (total 8 times). Tumor size was measured each week by a caliper. Tumor volume was calculated by height × major axis × minor axis × π/6. Mice were euthanized at 4 weeks after inoculation and subjected to histological examination.

### 4.14. Bone Marrow Replacement Model

BALB/c (5-week old, male, SLC) were euthanized by cervical dislocation. Bone marrow cells were harvested by flushing the bone marrow from the femur with regular DMEM (WAKO). Cells were resuspended in DMEM and pretreated with siMiro1 (10 nM, Sigma) or siControl (10 nM, Sigma) for 48 h according to manufacturer’s instructions. Bone marrow cells were then labeled with MitoLite Green EX488 (AAT Bioquest, Pleasanton, CA, USA), according to manufacturer’s instructions. The labeled cells were resuspended in HBSS (WAKO) at a volume of 1 × 10^7^ cells/0.25 mL. Subsequently, BALB/c (4-week old, male, SLC) recipient mice underwent 10 Gy of whole-body irradiation. In F-mice group, 5 mice were injected resuspended bone marrow cells (1 × 10^7^ per mouse) pretreated with siMiro1 from the tail vein. In C-mice group, 5 mice were injected bone marrow cells pretreated with siControl. CT26 cells (5 × 10^7^ per mouse) were inoculated into the scapular subcutaneous tissues at a week after bone marrow transplantation. Inoculated tumors were harvested from mice after euthanasia with anesthesia. Tumor tissues were cut into small pieces with a scalpel, treated with collagenase (Sigma) and DNAase (Sigma), and the isolated cells were transferred to a culture system with regular medium. Subsequently, the cultured cells were subjected to examinations.

### 4.15. Statistical Analysis

Statistical significance was calculated using a two-tailed Fisher’s exact test or an ordinary analysis of variance (ANOVA), using InStat software (GraphPad, Los Angeles, CA, USA). Correlations were tested using Pearson’s correlation test. A two-sided *p* value of <0.05 was considered to indicate statistical significance.

## Figures and Tables

**Table 1:** Primer sets, antibodies, and ELISA kits.

Gene Symbol	Species	Accession ID	Upper	Lower
*ACTB*	Mouse	NM_007393.5	agccatgtacgtagccatcc	ctctcagctgtggtggtgaa
*ACTB*	Human	NM_001101.3	ggacttcgagcaagagatgg	agcactgtgttggcgtacag
*GAPDH*	Human	BC025925.1	gagtcaacggatttggtcgt	ttgattttggagggatctcg
*GAPDH*	Mouse	NM_001289726.1	aactttggcattgtggaagg	acacattgggggtaggaaca
*Miro1*	Human	BC125105.1	cctgtactgcccagaggaga	ctgtcagccacaccatcact
*miro1*	Mouse	XM_021212699.2	ccggttacgctgcatgtgca	ggcaaagcccacaactgcga
*Cx43*	Human	M65188.1	atgagcagtctgcctttcgt	tctgcttcaagtgcatgtcc
*cx43*	Mouse	M63801.1	atcgcgtgaagggaagaagc	ctcgctggcttgcttgttgt
*Rage*	Human	AB036432.1	gctgtcagcatcagcatcat	attcagttctgcacgctcct
*Rage*	Mouse	L33412.1	aattgtggatcctgcctctg	aaggtaggatgggtggttcc
*TLR4*	Human	AB445638.1	cctgtccctgaaccctatga	ccagaaccaaacgatggact
*TLR4*	Mouse	AF177767.1	gctttcacctctgccttcac	gaaactgccatgtttgagca
*LGR5*	Mouse	NM_010195.2	cattcacttttggccgtttt	agggccaacaggacacatag
*LGR5*	Human	AF061444.1	ctcttcctcaaaccgtctgc	gatcggaggctaagcaactg
*Sox2*	Human	NM_003106.4	aaccccaagatgcacaactc	cggggccggtatttataatc
*CD44*	Human	FJ216964.1	aaggtggagcaaacacaacc	agctttttcttctgcccaca
*Klf4*	Human	KJ901962.1	cccacacaggtgagaaacct	cccacacaggtgagaaacct
*NS*	Mouse	BC037996.1	atgtggggaaaagcagtgtc	tgggggagttacaaggtgag
Antibodies				
Miro1	CL1083	ab188029	Abcam, Cambridge, MA, USA	
NFκBp65	D14E12	#8242	Cell Signaling Technology, Danvers, MA, USA	
HMGB1	3E8	651402	Biolegend, San Diego, CA, USA	
PARP	-	GTX132329	GeneTex, Irvine, CA, USA	
β-actin	C4	sc-47778	Santa Cruz Biotechnology, Santa Cruz, CA, USA	
GAPDH	1E6D9	60004-1-Ig	Proteintech, Tokyo, Japan	
ELISA				
Target	Species	Catalog number	Company	
CD74	Mouse	ELM-CD74-1	RayBiotech, Peachtree Corners, GA, USA	
SOX2	Mouse	LS-F14527	LS Bio, Shirley, MA, USA	
4HNE	-	ab238538	Abcam, Cambridge, MA, USA	

ACTB: β-actin; RAGE: receptor for advanced end products; TLR4: toll-like receptor 4; Miro1: mitochondrial Rho GTPase 1; Cx43: connexin 43; Sox2: sex determining region Y box 2; LGR5: leucine-rich repeat-containing G-protein coupled receptor 5; KLF4: Krüppel-like factor 4; GAPDH: glyceraldehyde 3-phosphate dehydrogenase; HMGB1: high mobility group box-1; PARP: poly ADP–ribose polymerase; NFκB: nuclear factor–κB; 4HNE: 4-hydroxynonenal.

## Data Availability

Data are contained within the article.

## Abbreviations

BM: bone marrow; MSC: mesenchymal stem cell; MT: mitochondrial transfer; CRC: colorectal cancer; 5-FU: 5-fluorouracil; HMGB1: anti-high mobility group box-1; rHMGB1: recombinant HMGB1; oxHMGB1: oxidized HMGB1; redHMGB1: reduced HMGB1; NF–κB: nuclear factor–κB, TME: tumor microenvironment; TNT: tunneling nanotubes; MMP: mitochondrial membrane potential; Miro1: mitochondrial Rho GTPase 1; Cx43: connexin 43; KD: knockdown; OXPHOS: oxidative phosphorylation.
